# Genome wide association study of body weight and feed efficiency traits in a commercial broiler chicken population, a re-visitation

**DOI:** 10.1038/s41598-018-37216-z

**Published:** 2019-01-29

**Authors:** Wossenie Mebratie, Henry Reyer, Klaus Wimmers, Henk Bovenhuis, Just Jensen

**Affiliations:** 1 Center for Quantitative Genetics and Genomics, Department of Molecular Biology and Genetics, Aarhus University, 8830 Tjele, Denmark; 2 Animal Breeding and Genomics Centre, Wageningen University, P.O. Box 338, 6700 AH Wageningen, The Netherlands; 3 Leibniz Institute for Farm Animal Biology, Institute for Genome Biology, Wilhelm-Stahl-Allee 2, 18196 Dummerstorf, Germany

## Abstract

Genome wide association study was conducted using a mixed linear model (MLM) approach that accounted for family structure to identify single nucleotide polymorphisms (SNPs) and candidate genes associated with body weight (BW) and feed efficiency (FE) traits in a broiler chicken population. The results of the MLM approach were compared with the results of a general linear model approach that does not take family structure in to account. In total, 11 quantitative trait loci (QTL) and 21 SNPs, were identified to be significantly associated with BW traits and 5 QTL and 5 SNPs were found associated with FE traits using MLM approach. Besides some overlaps between the results of the two GWAS approaches, there are considerable differences in the detected QTL. Even though the genomic inflation factor (λ) values indicate that there is no strong family structure in this population, using models that account for the existing family structure may reduce bias and increase accuracy of the estimated SNP effects in the association analysis. The SNPs and candidate genes identified in this study provide information on the genetic background of BW and FE traits in broiler chickens and might be used as prior information for genomic selection.

## Introduction

Genome wide association studies (GWAS) are commonly used to identify single nucleotide polymorphisms (SNPs) and candidate genes associated with quantitative traits. GWAS have revealed important regions associated with production, reproduction and disease resistance traits in chickens^1–7^.

One of the essential elements in GWAS is a powerful statistical method that can be employed to identify genetic associations in unbiased fashion^8^. Methods that model population structure by estimating the covariance due to genetic relatedness between individuals has been reported to perform better in terms of detecting true associations than models that ignore population structure^9–12^.

Kennedy *et al*.^13^ reported that using general linear model (GLM) analysis when relations between animals exist, results in an inflated F-test. Consequently it is likely to find an excess of spurious genotype effects when actually no genotype effect exists^13^. In this situation, the use of mixed-model procedures under an animal model treating single-gene effects as fixed effect and accounting for family relations can provide an exact F-test of associated hypotheses and unbiased estimates of genotype effects^13,14^.

Population structure mainly refers to population stratification and cryptic relatedness^15^. Population stratification is the presence of systematic differences in allele frequencies between subpopulations in a population due to different ancestry between study subjects^16^. Unrecognized population stratification can lead to both false positive and falsenegative findings and can obscure the true association signals if not appropriately corrected^17,18^.

Cryptic relatedness refers to the phenomenon that some members of a study sample (population) might be related beyond what can be inferred from the pedigree, in which case their genotypes are not independent of the population frequencies^19^. Because population based association studies assume individual independence of study samples, cryptic relatedness may make these statistical tests less reliable and reduce the robustness and efficiencies of the studies^15,17^.

Methods modeling population structure, family structure and cryptic relatedness are expected to perform better than models that ignore these complexities^11,15^. Mixed models offer a practical and comprehensive approach for simultaneously addressing confounding due to population stratification, family structure and cryptic relatedness^9,10,19^.

This study aims to identify potential loci and candidate genes associated with body weight (BW) and feed efficiency (FE) traits in a commercial broiler line genotyped with 60 k SNP chip using mixed linear model (MLM) approach that accounts for family structure and compare the results with general linear model (GLM) approach that does not take family structure in to account.

## Materials and Methods

### Ethical statement

Samples were collected from a commercial flock under the guidance of the local committees for the care and use of animals following the Cobb-Vantress Inc. Animal Welfare Policy. In addition, the experimental protocol was carried out in accordance with the approved guidelines for safeguarding good scientific practice at the institutions in the Leibniz Association.

### Birds and phenotypic data

Phenotypic data were obtained from Cobb-Vantress broiler breeding company. A total of 5000 male broilers fed standard commercial broiler chicken diet based mainly on maize were raised from hatch to five weeks of age. Only males were studied in this experiment since selection intensity in males is considerably higher than that of females in broiler breeding programs. At the age of 36 days, birds were weighed (BW36) and the heaviest 1000 birds were selected for feed efficiency (FE) experiment and put in individual cages. At the age of 39 days (BW39), birds entered to a 7 day FE experiment and final body weight (BW46) was recorded at the end of the experiment. Total feed intake (FI) was recorded from individually caged birds during the experiment. Body weight gain (Gain) was calculated as the difference between final body weight (BW49) and start weight (BW39). Feed conversion ratio (FCR) was calculated as the ratio of feed intake to body weight gain. After data cleaning, 848 BW and FE records were used for further analysis.

### Genotyping and quality control

Blood samples from the branchial vein were collected in anticoagulant tubes for DNA extraction. Extraction of genomic DNA was performed using Qiagen 96-well extraction kit (Qiagen, Hilden, Germany). DNA from a total of 864 samples were genotyped using the Illumina 60 K SNP chip (Illumina, San Diego, CA, USA). The Illumina 60 K SNP chip contains 57636 SNPs that are distributed across 29 autosomes (chromosome 1 to 28 and chromosome 32), two linkage groups (LGE 64 and LGE 22 C19 W28_E50C23), and two sex chromosomes (Z and W) using chicken genome assembly Galgal4. In this study only male broilers were examined so only genotypes from the Z sex chromosome were included. Plink software was used for quality control of the genotypic data^20^. SNPs with low call rates (<95%), minor allele frequency (MAF < 0.03) and Hardy Weinberg equilibrium (HWE) P-value (<0.0001) were excluded. After quality control a total of 43914 SNPs were retained for GWAS analysis.

### Genome wide association analysis

Genome wide association analysis was performed for BW traits (BW36, BW39, BW46) and for traits from the FE test (FI, Gain and FCR) using GCTA^21^. The genomic relationship matrix (GRM) was constructed using methods from Yang *et al*.^22^. The P-values were adjusted by Bonferroni correction based on linkage disequilibrium^23^. The effective number of independent SNPs of autosomes were defined by the independent pairwise option in plink^20^. A total of 19416 independent SNPs were identified and the 5% genome wide significance threshold was adjusted to -log10 (P-value) = 5.60. The threshold P-value for suggestive significant association that allows one false positive association per GWAS was adjusted to -log10 (P-value) = 4.3. Genomic inflation factor (λ), was calculated using the R package GenABEL with “median” option^24^.

Manhattan plots of genome wide association analysis and quantile-quantile (QQ) plots were created using the qqman package in R software^25^. The annotated genes that were closest to the top SNPs were identified using Ensembl and NCBI. The reported top SNPs or “lead SNPs” are SNPs which have the highest -log 10 (P-value) among the significant SNPs which are in linkage disequilibrium (LD) with each other in 1 Mb windows. Base pair positions of SNP markers were updated to the latest version of the chicken genome assembly Gallus-gallus-5 (Galgal5).

For FI and Gain, the following linear mixed model was used:

1 $${\rm{y}}=\mu +{{\rm{\beta }}m}_{{\rm{i}}}+{{\rm{\beta }}}_{1}{\rm{x}}+{\rm{Zu}}+{\rm{e}}$$

For FCR and BW traits the following linear mixed model was used:

2 $${\rm{y}}=\mu +{{\rm{\beta }}m}_{{\rm{i}}}+{\rm{Zu}}+{\rm{e}}$$

Where, y is a vector of BW or FE observations, μ is the mean term, β is the SNP effect for marker i, m is a vector of SNPs for the i^th^ SNP genotype indicator variable coded as 0, 1 or 2, β_1_ is the regression coefficient (the effect of start weight on FI and Gain that accounts for differences in start weight), x is a vector of start weights (BW39), u is a vector of random polygenic effects i.e. The effect of all QTL except those on the chromosome where the candidate SNP is located, e is a vector of random residuals. The variance of u was re-estimated each time when a chromosome was excluded from calculating the genetic relationship matrix. Z is the incidence matrix for the random effect. The variance co-variance structure for the random effects were assumed to be normally distributed with mean 0 and variance; var (u) = ${\bf{G}}{{\rm{\sigma }}}_{{\rm{g}}}^{2}$ and var (e) = ${\bf{I}}{{\rm{\sigma }}}_{{\rm{e}}}^{2}$. Start weight was included in the model as covariate for FI and Gain in order to account for differences in start weight for these traits. The genomic relationship matrix of this broiler population indicated that there are only small number of half sibs and full sibs in the data^26^, therefore maternal effects were not included in the model.

### Comparison of MLM and GLM approaches

The results of the present study were compared with the results of the GLM analysis by Reyer *et al*.^5^ which does not take family structure in to account, using the following model.

3 $${\rm{y}}=\mu +{{\rm{\beta }}m}_{i}+{{\rm{\beta }}}_{1}{\rm{x}}+{\rm{e}}$$

Where, y is the BW or FE observations, μ is the mean term, β is the SNP effect for marker i, m is vector of markers for the i^th^ SNP genotype indicator variable coded as 0, 1 or 2, β_1_ is the regression coefficient (the effect of start weight on FI and Gain) that accounts for differences in start weight, x is a vector of start weights (BW39) for FI and Gain, and, e is the random residual. The variance of e is I${{\rm{\sigma }}}_{{\rm{e}}}^{2}$. Base pair positions of SNP markers in Reyer *et al*.^5^ were updated to the latest version of the chicken genome assembly, Gallus-gallus-5 (Galgal5) for easy comparison of the results with the current study.

## Results

### GWAS results for body weight traits

**Figure 1:**
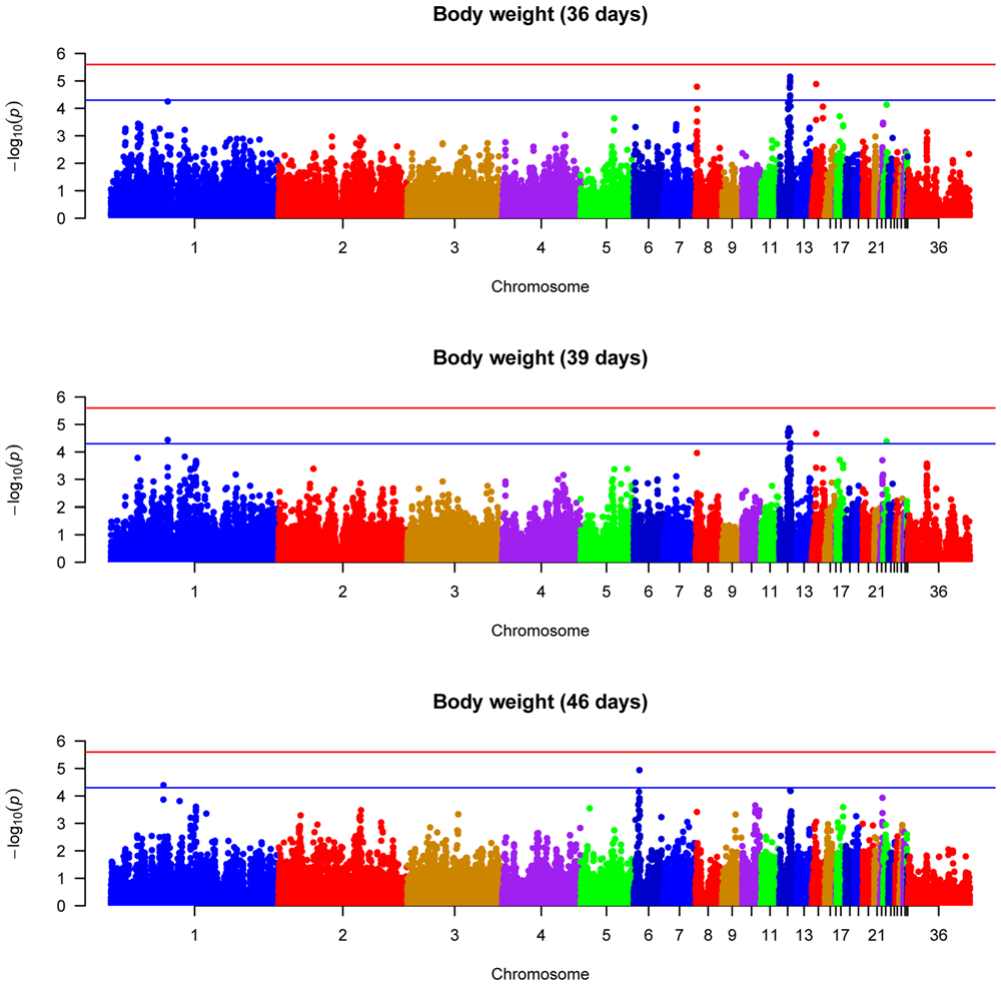
Manhattan plots of genome wide association results for body weight traits using mixed linear model analysis. Chromosomes 29, 30 and 36 represent linkage groups LGE22C19W28_E50C23, LGE64, and chromosome Z, respectively. Red and blue lines indicate genome wide and suggestive significance thresholds, respectively.

**Figure 2:**
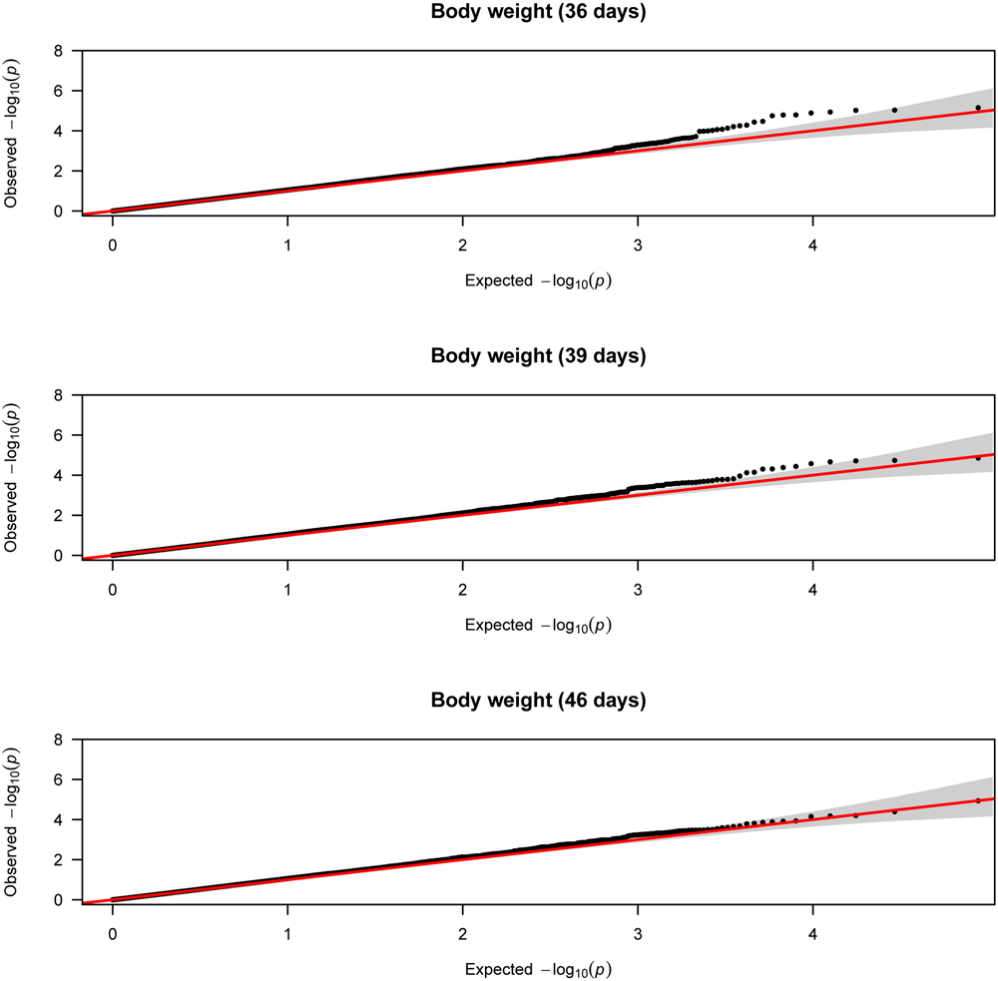
Quantile-quantile plots of body weight traits using mixed linear model approach.

Using MLM approach, the present study revealed 3 QTL which have suggestive significance association with BW36, 6 QTL with BW39 and 2 QTL with BW46 (Table 1). A total of 11 QTL and 21 SNPs reached the suggestive significance level with BW traits. The top SNPs and candidate genes associated with BW36 are located on chromosome 12, 14, and 8 while the top SNPs associated with BW39 are located on chromosome 12, 14, 1 and 23. The top SNPs associated with BW 46 are located on chromosome 6 and 1 (Table 1). Manhattan plots and QQ plots of body weight traits are shown in Figs 1 and 2, respectively. All the reported QTL in Table 1 are suggestive, no SNP reached the genome-wide significance level for BW and FE traits in the present study, which suggests that BW and FE traits are controlled by many genes, each with small effects. All of the reported top SNPs were found inside the candidate genes in an intronic region except SNP rs15652523 associated with BW39, which is, located 2.52 Kb upstream of the candidate gene LOC107054392 (Table 1).

**Table 1:** Top SNPs associated with body weight and feed efficiency traits using mixed linear model approach.

Trait	Chromosome	Number of significant SNPs	Top SNP in 1 MB window	Galgal5 position (bp)	-log10 (P-value)	SNP effect (SE)	Proximal gene	Distance from gene
Body weight (36days)	12	8	rs13612706	12867052	5.03	−0.039 (0.009)	PTPRG	Within
Body weight (36 days)	14	1	rs14073523	5337950	4.89	0.036 (0.008)	CACNA1H	Within
Body weight (36 days)	8	1	rs16617885	1883743	4.79	−0.051 (0.012)	LOC107053920	Within
Body weight (39 days)	12	1	rs10723005	11570033	4.89	−0.054 (0.012)	CCDC71	Within
Body weight (39 days)	12	3	rs316610173	12931647	4.74	−0.047 (0.011)	PTPRG	Within
Body weight (39 days)	12	2	rs15652523	10278318	4.72	−0.043 (0.010)	LOC107054392	2.52 Kb upstream
Body weight (39 days)	14	1	rs14073523	5337950	4.67	0.041 (0.010)	CACNA1H	Within
Body weight (39 days)	1	1	rs13880135	66076666	4.44	0.040 (0.010)	SOX5	Within
Body weight (39 days)	23	1	rs16190017	3741996	4.38	0.043 (0.011)	RSPO1	Within
Body weight (46 days)	6	1	rs315083186	7305184	4.94	−0.118 (0.027)	LOC101748440	Within
Body weight (46 days)	1	1	rs314956606	61033904	4.39	−0.095 (0.023)	ADIPOR2	Within
Feed intake	1	1	rs15384287	110928416	4.96	−0.111 (0.021)	KDM6A	Within
Body weight gain	8	1	rs16617885	1883743	4.43	−0.058 (0.013)	LOC107053920	Within
Body weight gain	17	1	rs14098962	7902999	4.32	−0.054 (0.012)	LOC107052218	Within
Feed conversion ratio	17	1	rs14098962	7902999	4.78	0.082 (0.019)	LOC107052218	Within
Feed conversion ratio	6	1	rs14568465	6730175	4.30	0.073 (0.018)	CTNNA3	Within

SNP, rs14042911, is one of the top SNPs (-log 10 (P-value) = 5.15) associated with BW36 which is located on chromosome 12. Due to the proximal position to the top SNP, PTPRG (protein tyrosine phosphatase, receptor type G) is proposed as candidate gene associated with BW36 (Table 1). PTPRG is a protein coding gene which is a member of the protein tyrosine phosphatase (PTP) family. In humans members of the PTP gene family are known to be signaling molecules that regulate a variety of cellular processes including cell growth and differentiation, mitotic cycle, and oncogenic transformation^27^. Moreover, SNPs, rs14073523 and rs16617885, located on chromosome 14 and 8, are top SNPs associated with BW36. CACNA1H (calcium voltage-gated channel subunit alpha1 H); a protein coding gene and LOC107053920 (uncharacterized gene) are the proximal genes to the top SNPs, rs14073523, and rs16617885, respectively. Wang *et al*.^2^ identified QTL located on chromosome 14 associated with abdominal fat weight in chickens. The detected QTL for BW traits in the current study suggest that the genetic variance of BW was not exhausted after the pre-selection rather there are many genes with small effects left in the population. This is a remarkable finding relative to our expectation given the fact that this broiler line has been pre-selected for BW. Mebratie *et al*.^26^ also noted an increase in genetic variance of BW after several generations of selection in a commercial broiler chicken population.

This study also revealed 6 QTL located on chromosome 12, 14, 1 and 23, to have suggestive significance association with BW39, 3 of them located on chromosome 12. SNP, rs10723005, is one of the top SNPs, associated with BW39 found on chromosome 12 which is located in an intron region of CCDC71 (coiled-coil domain containing 71 recombinant protein). SNPs, rs316610173, and, rs15652523, which are located on chromosome 12 and SNPs, rs14073523, rs13880135, and, rs16190017, located on chromosome 14, 1 and 23, respectively, are also found associated with BW 39 (Table 1). PTPRG, LOC107054435 (uncharacterized gene), CACNA1H, SOX5 (transcription factor SOX-5) and RSPO1 (R-spondin 1) are candidate genes, with putative contribution to the variation in BW39. They are found proximal to SNPs, rs316610173, rs14073523, rs13880135 and rs16190017, respectively (Table 1). In humans, SOX5 is involved in the regulation of embryonic development and in the determination of the cell fate^27^ while RSPO1 gene in mice is involved in the rapid onset of crypt cell proliferation^27^.

Two QTL located on chromosome 6 and 1, showing suggestive significance association with BW46 were identified with MLM approach. SNP, rs315083186, located on chromosome 6 and, SNP, rs314956606, located on chromosome 1 were found significantly associated with BW46 (Table 1). The candidate genes associated with BW46 are LOC101748440 (uncharacterized gene) and ADIPOR2 (adiponectin receptor 2), which is a protein coding gene involved in fatty acid oxidation and glucose uptake in humans^27^. In the chicken QTL database^28^, 3 QTL located on chromosome 12 and 2 QTL located on chromosome 12 are reported to be associated with BW36 and BW46, respectively. One of the 3 QTL reported in the chicken QTL database for BW36 by Reyer *et al*.^5^ is overlapping with the identified QTL in the current study. However, the others are different QTL on the same chromosome, suggesting that chromosome 12 is potential chromosome for QTL associated with BW traits.

### GWAS results for feed efficiency traits

**Figure 3:**
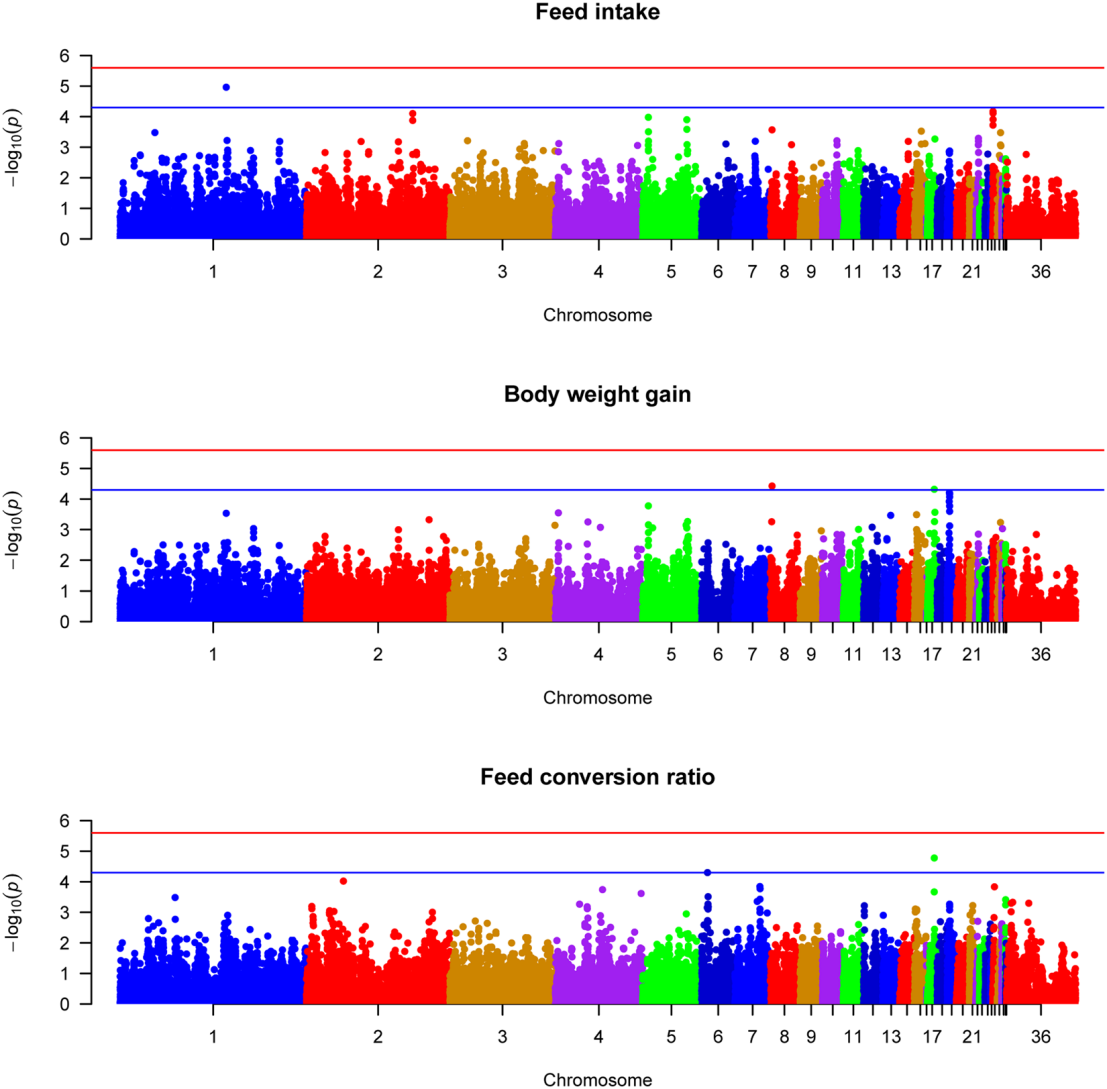
Manhattan plots of genome wide association results for feed efficiency traits using mixed linear model analysis. Chromosomes 29, 30 and 36 represent linkage groups LGE22C19W28_E50C23, LGE64, and chromosome Z, respectively. Red and blue lines indicate genome wide and suggestive significance thresholds, respectively.

**Figure 4:**
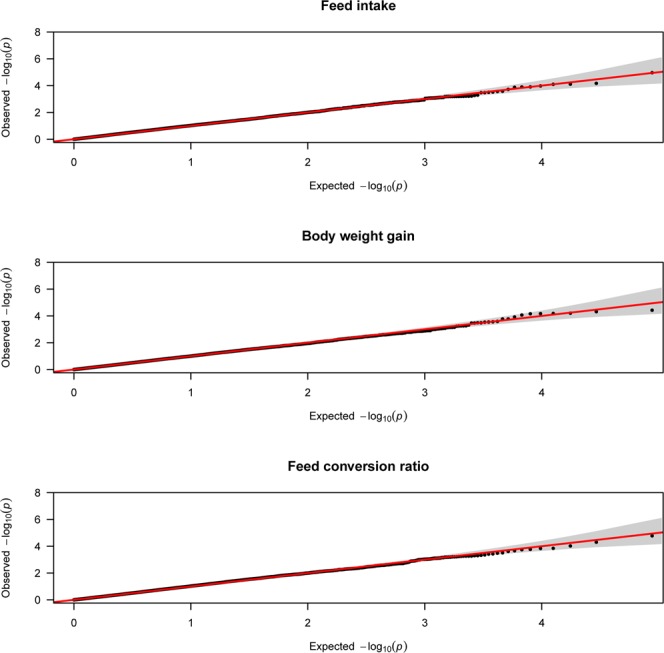
Quantile-quantile plots of feed efficiency traits using mixed linear  model approach.

In this study a total of 5 QTL and 5 SNPs that showed suggestive significance association with FE traits were identified using MLM approach. QTL located on chromosome 1 is found associated with feed intake while 2 QTL located on chromosome 8 and 17 are found associated with body weight gain. Two QTL located on chromosome 17 and 6 are found significantly associated with FCR. The top SNPs and proximal genes associated with FI, Gain and FCR are reported in Table 1. Manhattan plots and QQ plots of feed efficiency traits are shown in Figs 3 and 4, respectively.

QTL that contain SNP, rs15384287, located on chromosome 1 is found to be associated with feed intake (Table 1). The candidate gene associated with feed intake is KDM6A (lysine demethylase 6A). In line with this study, Yuan *et al*.^7^ reported a region on chromosome 1 that contains 8 SNPs which are significantly associated with feed intake in laying hens. Similarly, Mignon-Grasteau *et al*.^29^ reported four QTL associated with feed intake on chromosome 1 in chickens. Gao *et al*.^30^ and Tran *et al*.^31^ also reported QTL located on chromosome 1 to be associated with Gizzard weight in chickens which might have positive correlation with feed intake.

Two QTL located on chromosome 8 and 17 reached the suggestive significance level with body weight gain. SNP, rs16617885, located on chromosome 8 and SNP, rs14098962, located on chromosome 17 are found associated with body weight gain (Table 1). LOC107053920 and LOC107052218 are uncharacterized candidate genes associated with the trait which are found proximal to the significant SNPs, rs16617885 and rs14098962, respectively. SNP, rs16617885, located on chromosome 8 is also found associated with BW36 with overlapping uncharacterized candidate gene, LOC107053920 (Table 1).

Two QTL located on chromosome 17 and 6 showed suggestive significance association with FCR. SNPs, rs14098962 and rs14568465, located on chromosome 17 and 6, respectively are found associated with FCR (Table 1). CTNNA3 (catenin alpha 3) and LOC107052218 (uncharacterized gene) are the candidate genes found proximal to SNPs, rs14098962 and rs14568465, respectively. SNP, rs14098962, located on chromosome 17 and candidate gene LOC107052218 are also found associated with body weight gain (Table 1). Yuan *et al*.^7^ reported a region on chromosome 17 which is significantly associated with residual feed intake (RFI) in laying hens. In the chicken QTL database^28^ different authors have reported different QTL on chromosome 1 to be associated with FI. Similarly QTL on chromosomes 6 and 17 were reported to be associated with FCR in the QTL database. The reported chromosomes are overlapping with the chromosomes associated with FI and FCR in the current study. However, the QTL regions are different except the QTL reported by Reyer *et al*.^5^ for FCR located on chromosome 6 which were identified using Bayesian method of analysis for the same broiler line.

### Comparison of results in MLM and GLM approach

The results of the MLM analysis in this study were compared with the results of the GLM analysis by Reyer *et al*.^5^ (Table 2). There are some overlaps between the identified QTL and candidate genes for BW36 and FCR. However, for most of the BW and FE traits, the identified QTL and candidate genes were different suggesting that the two methods do not necessarily give similar results. Table 3 shows comparison of genomic inflation factor (λ) between MLM and GLM analysis. The λ values in both approaches are “benign” and not significantly different from each other suggesting that population stratification is not a strong concern in our data.

**Table 2:** Top SNPs associated with body weight and feed efficiency traits using general linear model approach by Reyer *et al*. (2015).

Trait	Chromosome	Number of significant SNPs	Top SNP in 1MB window	Galgal5 position (bp)	-log10 (P-value)	Candidate gene
Body weight (36 days)	12	8	rs13612706	12867052	5.31	PTPRG
Body weight (36 days)	14	1	rs14073523	5337950	4.42	CACNA1H
Body weight (46 days)	8	1	rs16617885	1883740	5.21	PTPRC,NR5A2
Body weight (46 days)	Z	1	rs14753816	19805476	4.89	HTR1A
Feed intake	5	1	rs16266739	6549732	4.38	SPON1
Feed intake	19	1	GGaluGA001282 (rs313913143)	1878406	4.61	ENSGALG0000002830
Feed intake	26	1	rs15467593	539371	4.50	KDM5b
Body weight gain	17	1	GGaluGA117403 (rs312843163)	9904101	5.29	GPR144,NR5A1,NR6A1
Feed conversion ratio	4	2	rs14445503	31108872	5.04	HHIP
Feed conversion ratio	17	1	GGaluGA117403 (rs312843163)	9904101	6.57	NR5A1, NR6A1
Feed conversion ratio	17	1	rs14098962	7902999	4.86	RXRA
Feed conversion ratio	22	1	GGaluGA186837 (rs312757200)	4533949	5.07	ADRA1A

The position of SNPs is updated to the latest chicken genome assembly (Galgal5).

**Table 3:** Comparison of lambda values with standard errors in general linear model (GLM) and mixed linear model (MLM) analysis.

Trait	Lambda GLM	SE	Lambda MLM	SE
Body weight (36 days)	1.0812	9.921616e-05	1.0532	0.0001531706
Body weight (39 days)	1.0925	8.277596e-05	1.0423	8.860991e-05
Body weight (46 days)	1.0592	0.002739952	1.0057	0.0001031297
Feed intake	1.0429	8.538701e-05	1.0242	0.0001203372
Body weight gain	1.0022	6.283618e-05	0.9955	9.700197e-05
Feed conversion ratio	1.0513	0.0002345801	1.0258	0.0001107539

Reyer *et al*.^5^ have reported GWAS results for two BW traits (BW36 and BW46) and three FE traits (FI, Gain and FCR) using the same data. Table 2 shows the reported GWAS results of the BW and FE traits derived from a general linear model (GLM) by Reyer *et al*.^5^. The GLM analysis was replicated in this study to compute the λ values reported in Table 3 and we found similar significant SNPs and -log 10 (P-values) for all of the BW and FE traits as reported by Reyer *et al*.^5^ (Table 2). BW39 was not considered in the analysis of Reyer *et al*.^5^ but considered both in the replicated GLM analysis and the MLM analysis in this study.

Using the GLM approach, Reyer *et al*.^5^ reported 2 QTL and 9 SNPs that reached the suggestive significance level, located on chromosome 12 and 14 which were associated with BW36. The reported top SNPs, rs13612706, rs14073523, and proximal candidate genes associated with BW 36 are consistent with the present study (Tables 1 and 2). SNP, rs13612706, showed higher -log 10 (P-value) in the GLM analysis compared to the MLM approach, whereas SNP, rs14073523, was indicated by a higher -log 10 (P-value) in the MLM approach than the GLM approach (Tables 1 and 2). In both MLM and GLM approaches 8 SNPs located on chromosome 12 were found significantly associated with BW36 with overlapping candidate gene, PTPRG (Tables 1 and 2). All the reported QTL in Reyer *et al*.^5^ are those that reached the suggestive significance level (-log10 (P-value) ≥ 4.3). No QTL reached genome wide significance level (-log10 (P-value) ≥ 5.6) except QTL on chromosome 17 which is associated with FCR (Table 2).

Reyer *et al*.^5^ also reported 2 QTL and 2 SNPs associated with BW46 located on chromosome 8 and Z, 1 QTL and a single SNP associated with body weight gain located on chromosome 17, 1 QTL and 3 SNPs associated with feed intake located on chromosome 5, 19 and 26, which are not consistent with the present study (Table 2). Moreover, Reyer *et al*.^5^ reported 4 QTL and 5 SNPs which have significant association with FCR, 2 of them located on chromosome 17, 1 located on chromosome 4 and the remaining QTL located on chromosome 22 (Table 2). Among the reported top SNPs associated with FCR, SNP rs4098962, located on chromosome 17 was found overlapping with the present study with higher -log 10 (P-value) in the GLM approach compared to the MLM approach. However, the reported candidate genes are different (Tables 1 and 2).

For most of the BW and FE traits, the reported QTL in this study are not consistent with the reported QTL by Reyer *et al*.^5^. A total of 12 QTL and 20 SNPs associated with two BW (4 QTL and 11 SNPs) and three FE traits (8 QTL and 9 SNPs), were reported by Reyer *et al*.^5^ using the GLM approach (Table 2). By applying the MLM approach, the present study identified a total of 10 QTL and 17 SNPs, associated with the same BW (5 QTL and 12 SNPs) and FE (5 QTL and 5 SNPs) traits (Table 1). Among the identified 10 QTL in the MLM approach only 2 QTL associated with BW36 and FCR were found overlapping with the GLM approach while the 8 identified QTL were different from the ones reported in Reyer *et al*.^5^ for the same BW and FE traits.

## Discussion

Body weight and feed efficiency traits are the most important economic traits in the poultry industry. Body weight is the live weight of birds at a given age and feed efficiency (FE) is the ability of birds to convert a certain input to a certain output (e.g. Kg of feed in to Kg of meat)^32^. Among the number of ways to asses feed efficiency, the most widely used are feed conversion ratio (FCR) and residual feed intake^32^.

We have performed GWAS for body weight and feed efficiency traits in a commercial broiler chicken population using a MLM approach, taking family structure into account. The results were compared with the results of a GLM approach, which does not take family structure into account.

Reyer *et al*.^5^ also used Bayesian (Multi-marker) approach, which is more robust to population stratification^33^ and reported more significant SNPs associated with BW and FE traits than the GLM approach. However, in this paper the results  were only compared with the single marker GLM approach which is comparable to the single marker MLM approach in the present study.

Xu *et al*.^6^ reported that chromosome 1 and 4 are the two critical chromosomes influencing growth traits particularly body weight in chickens. In this study, SNPs on chromosome 1 were found to be associated with BW39 and BW46 while no significant SNP were found on chromosome 4 for any of the BW traits under study. Podisi *et al*.^34^ also reported two significant QTL for body weight at 12 weeks of age on chromosome 1 in broiler-layer cross female chickens.

Some of the BW and FE traits share consistent QTL and candidate genes. BW36 and BW39 share consistent region on chromosome 14 and candidate genes, PTPRG and CACNA1H, while BW46 does not share those candidate genes with BW36 and BW39. The effects of the lead SNPs with standard errors in parenthesis for BW36 and BW39 were found to be 0.036 (0.008) and 0.041 (0.010), respectively. This might indicate that the effects of these genes are smaller on BW46 due to increasing importance of other genes for the trait, suggesting that the identified genes are age dependent and the two traits (BW36 and BW39) might be genetically correlated. Mebratie *et al*.^26^ have reported that the genetic correlation between BW at different ages increased as the distance between BW measurements decreased. This might be due to changes in the physiological system of the chickens with age. Indeed, Schaeffer^35^ states that there might be genes that “switch on” and “off” at a certain age of an animal which could lead to changes in physiology and performance. Similarly, Carlborg *et al*.^36^ concluded that there are different genes and gene actions involved in growth at different developmental stages.

BW36 and body weight gain share an overlapping region on chromosome 8 and consistent candidate gene, LOC107053920, with SNP effects −0.051 (0.012) and −0.058 (0.013), respectively (Table 1). This might suggest high positive genetic correlation between BW36 and body weight gain which is not surprising since body weight gain is a component of body weight. Furthermore, body weight gain and FCR share consistent QTL on chromosome 17 and candidate gene LOC107052218 (Table 1) with SNP effects −0.054 (0.012) and 0.082 (0.019), respectively.

This broiler line is pre-selected for BW (only heaviest birds were entered to the FE experiment) and undergone several generations of selection for feed efficiency. This phenomenon might affect the detection power of our GWAS and estimated SNP effect sizes since the genetic variance of BW and FE traits might be reduced due to the pre-selection and several generations of intense selection. Mebratie *et al*.^37^ have reported that the SNP based estimated genetic variance of BW (0–0.006 kg^2^) and FE traits (0.001–0.006 kg^2^) in this broiler population is very small with high standard error and among others, one of the reasons for the reported very small estimates of genetic variance was pre-selection of the broiler line for BW.

We have conducted GWAS using MLM approach, which takes in to account family structure and compared the results with the GLM approach by Reyer *et al*.^5^ which does not take family structure in to account. A number of studies^38,39^ have shown that methods that model family structure perform better than models that ignore family structure. A widely used approach to evaluate whether confounding due to population stratification, family structure and cryptic relatedness exists is to compute the genomic inflation factor (λ), which is defined as the median χ2 (1 degree of freedom) association statistic across SNPs divided by its theoretical median under the null distribution^40^. Values of genomic inflation factor (λ) > 1 generally indicate population stratification or other confounders, such as family structure or cryptic relatedness^19^. Values of λ < 1.05 are considered “benign” regarding power and type I error^19,41^, although inflation in λ is proportional to sample size.

Table 3 shows the λ values of BW and FE traits using the MLM approach in the present study and the GLM approach by Reyer *et al*.^5^. The genomic inflation factor values suggest that population structure is not a strong concern in our data and the values are not significantly different from each other in the two methods. Moreover, the genomic relationship matrix of individuals shows that there are only few half sib and full sib relations in the current data^38^ suggesting that family structure is not a strong concern. However, there is a slightly higher inflation of λ values in the results of Reyer *et al*.^5^ compared to the results of the present study. This may suggest that although there is no strong family structure in the population, using MLM analysis that takes in to account the existing family structure may increase power to detect true associations than ignoring this kind of sample structure which may result spurious associations.

In a simulation study, Thornton *et al*.^42^ have noted that in the absence of markers with unusual allele frequency differences (markers with allele frequency differences that lie outside the expected distribution which could be caused by natural selection) using the genomic relationship matrix to account for both population and family structure can effectively control spurious associations under a variety of settings. Price *et al*.^19^ have also suggested that in studies where population stratification is not a very serious concern, an appealing and simple approach is to use mixed models.

For the commercial broiler chicken data used in this study, family structure is not a strong concern. However, as suggested by different authors^14,19,42^, we have used a MLM approach that takes in to account the existing family structure and revealed differences in the identified top SNPs and candidate genes associated with BW and FE traits compared with the GLM approach by Reyer *et al*.^5^. This strengthens the suggestion that, even though there is no strong family structure in the data, MLM approach that uses the genomic relationship matrix to account for the existing family structure may decrease bias and improve accuracy of the association analysis.

The results of this study might provide insight about the genetic background of body weight and feed efficiency traits. Furthermore, the study emphasizes that GWAS using the two approaches (GLM and MLM) does not necessarily give similar results even with the absence of strong family structure in the data.

## Conclusions

GWAS for BW and FE traits was performed in a commercial broiler chicken population. The present study has identified 11 QTL and 21 SNPs associated with BW traits and 5 QTL and 5 SNPs associated with FE traits. The results of this study provide insight on QTL and genes that are involved in the genetics of BW and FE traits in broiler chickens and can be used as fundamental information for genomic selection. Moreover, the MLM approach, which takes in to account the existing family structure by using the genomic relationship matrix, resulted in different QTL for most of the analyzed BW and FE traits compared to the GLM approach that ignored the existing family structure. Although, there is no strong family structure in this population, the use of MLM approach may increase power to detect true associations compared to the GLM approach that does not take family structure into account as suggested by previous studies.

## Data Availability

Data supporting this paper were obtained from Cobb-Vantress chicken breeding company. The phenotype and genotype data are available only upon agreement with Cobb-Vantress and should be requested directly from the breeding company.
